# Factors associated with depressive symptoms in patients with ankylosing spondylitis in Northern Taiwan

**DOI:** 10.1371/journal.pone.0224298

**Published:** 2019-10-24

**Authors:** Mei-Ling Fang, Chien-Sheng Wu, Li-Chueh Weng, Hsiu-Li Huang

**Affiliations:** 1 Department of Nursing, Far Eastern Memorial Hospital, New Taipei City, Taiwan; 2 Division of Allergy, Immunology and Rheumatology, Far Eastern Memorial Hospital, New Taipei City, Taiwan; 3 School of Nursing, College of Medicine, Chang Gung University, Taoyuan, Taiwan; 4 Department of Long-term Care, College of Health Technology, National Taipei University of Nursing and Health Sciences, Taipei, Taiwan; University of New South Wales, AUSTRALIA

## Abstract

Patients with ankylosing spondylitis (AS) experience impaired physical function and reduced quality of life, which puts this group at high risk for depression. Identifying factors associated with depressive symptoms could improve outcomes for this at-risk group. However, few studies have examined the relationship between demographic and clinical variables and depressive symptoms in patients with AS. This cross-sectional correlation study recruited patients with AS by convenience sampling from the division of immunology and rheumatology of a medical center in Northern Taiwan. Participants (N = 120) included 91 males and 29 females, age ≥ 20 years. Data were collected from chart reviews, and structural questionnaires, which included demographic information regarding employment status, history of falls, impact of AS on work; clinical information relative to AS was obtained from structural questionnaires: the Bath Ankylosing Spondylitis Disease Activity Index (BASDAI) and functional index (BASFI), Numerical Rating Scale (NRS), Body Image Scale (BIS), and Beck Depression Inventory-II (BDI-II). Multiple regression analysis identified predictors of depression. The mean BDI-II score was 9.50 ± 8.30; 25% had scores indicating mild to severe depressive symptoms. Mean score on the BIS was 68.17 ± 16.14; 14.2% had fallen within the previous year; and 57.5% reported AS affected their work. Variables associated with depressive symptoms were work affected by AS (*β* = 0.14, *p* = .049), occurrence of a fall within the previous year (*β* = 0.14, *p* = .032), higher scores on the BASDAI (*β* = 0.21, *p* = .032), and lower body image (*β* = −0.38, *p* < .001). Clinical professionals should regularly assess patients with AS for depressive symptoms. Health care planning should provide instruction in fall prevention and control of disease activity, and strategies to improve body image, which could improve patients’ self-management capabilities and body image as well as mitigate depressive symptoms.

## Introduction

Ankylosing spondylitis (AS) is a progressive autoimmune disease that primarily affects the axial skeleton; joint cavities replaced by newly formed bones cause spinal deformities and sacroiliac joint diseases, which leads to pain, abnormal posture, and movement disorders [1, 2]. The prevalence of AS varies by sex and ethnicity; males are 1.2–7 times more likely to be diagnosed with AS than females; the prevalence per 10,000 is higher for Europeans and Asians (23.8 and 16.7 respectively) than for people in Latin America and Africa (10.2 and 7.4, respectively) [3]. An estimated 4.63–4.98 million people in Asia have been diagnosed with AS [3]. In Taiwan, the reported prevalence is 0.10%, and diagnosis peaks at 20–29 years of age [4]; a younger the age at onset of AS is correlated with an unfavorable prognosis [4, 5].

Symptoms of AS affect patients’ functions and activities of daily living, as well as ability to work. Patients’ psychological functions are also influenced by AS; the changes in physical appearance associated with AS cause patients to be more focused on body image than healthy individuals, resulting in body image disturbance, and lower self-worth[6, 7]. Thus, the impact of AS on physical and emotional functions may explain the comorbidity of depression that is common among patients with AS [8–10].

The prevalence of depression among patients with AS is higher than that among healthy individuals [6, 11–13]. However, correlations between variables for patients with AS and depression vary widely. A systematic literature review by Zhao et al. (2018) on depression in patients with AS found a large disparity in prevalence of depression (11%–64%), as well as correlations of sex, disease activity, and functional impairment associated with depression [14].

Depression in patients with AS is significantly correlated with measures of pathophysiology, such as the Bath Ankylosing Spondylitis Disease Activity Index (BASDAI), the Bath Ankylosing Spondylitis Functional Index (BASFI), and indicators of pain and inflammation such as erythrocyte sedimentation rate (ESR) and C-reactive protein (CRP) [10, 14–16]. However, the causes of depression are complex, and despite its prevalence among patients with AS, depression is frequently overlooked during clinical care [14]. Depression is often the result of perception of body image due to skeletal changes that accompany AS [7]; poorer body image not only results in depression, but also a below-average quality of life [7, 17–19].

Depression is also exacerbated by the external stress of juggling AS symptoms and work expectations; after adjusting for age and sex, unemployment and work instability was found to be three times higher among patients with AS than among healthy individuals [20]. Depression, fatigue, and work instability scores are significantly higher in patients with AS than controls [21, 22]; work instability, number of hours of work, and job changes are often a result of AS [22]. However, few studies have examined the correlation between depression and working status among patients with AS [23].

Although patients with AS experience depression in addition to long-term physical symptoms, few studies have comprehensively examined the relationship between depression and physical, psychological, and social factors such as disease progression, falls, body image, and work-related factors [22, 24, 25]. The aim of this study was to evaluate levels of depressive symptoms in patients with AS and identify factors associated with depressive symptoms, thus providing a reference for implementing clinical care interventions for patients with AS.

## Materials and methods

### Participants

Patients for this study were recruited from an immunology and rheumatology division of a medical center in northern Taiwan. Patients were included if they met the following inclusion criteria: aged 20 years or older, which is based on the Taiwan Human Subjects Research Act [26], which defines the age of consent as 20 years old; a confirmed diagnosis of AS, which followed the modified New York criteria [27]; exhibited clear consciousness; able to communicate in Mandarin or Taiwanese; agreed to answer the survey questionnaire; and consented to collection of data from their medical charts. Patients were excluded if they had a history of being diagnosed with infectious diseases, spinal tumors, cancer, or dementia.

The number of participants necessary was estimated using the regression model of G*Power 3.1 [28]. We also applied the method described by Grove et al. (2013) for determining a correlation value equal to the effect size for the relationship between two variables [29]. Findings from a previous study estimated the correlation coefficient between disease activity and depressive symptoms and between body image and depressive symptoms in a rheumatoid arthritis population to be r = 0.19 and r = 0.59, respectively [30]. We chose the lower correlation coefficient, and set the effect size at 0.19, 90% power, and an α level of 0.05. The number of patients required for 10 predictor variables was estimated to be 120.

This study was written in accordance with the Declaration of Helsinki and approved by the Far Eastern Memorial Hospital Research Ethics Review Committee (FEMN-105080-E). The researchers personally explained the purpose, steps, benefits, and risks of this study, and assured patients of anonymity of their data. All participants provided written informed consent before the questionnaire survey was administered and medical data was collected from patient charts. Participants who were unable to complete the questionnaire on site were provided with return envelopes and were instructed to return their completed responses within three days. Responses were anonymous and encoded to safeguard the participants’ privacy.

### Data collection and measurements

Data were collected from January to December, 2017. Physio-pathological data were collected from patient charts. A structural questionnaire was used to collect data regarding demographic information, and measurement scales regarding disease activity, functions of daily living, pain, body image, and depression. Because of financial and geographic limitations, only the data from the immunology and rheumatology division of a medical center in the Northern Taiwan were collected.

#### Physio-pathological data

For the purpose of clinical measures associated with AS, data were collected from patients’ charts. For determining inflammation status values for c-reactive protein (CRP) and erythrocyte sedimentation rate (ESR) were retrieved for the preceding six months [31]. The severity of sacroiliac joint dysfunction and Cobb’s angles were measured by the attending physician through x-ray interpretation and measurement. Sacroiliac joint dysfunction severity was separated into the following levels. (1) Level 0: no deformity; (2) Level 1: deformity suspected; (3) Level 2: mild deformity, sclerosis, and partial erosion; (4) Level 3: moderate deformity, severe erosion, pseudo-widening of joints, and partial ankylosis; and (5) Level 4: complete ankylosis [32]. A higher level indicated more severe sacroiliac joint dysfunction. Cobb’s angles were divided into the following levels: (1) normal angle (0°–10°); (2) mild kyphosis (11°–20°); (3) moderate kyphosis (21°–40°); and (4) severe kyphosis (> 40°). A larger Cobb’s angle indicated more severe thoracic and lumbar kyphosis [32].

#### Demographic data

The items pertaining to demographic information were self-developed according to relevant literature and included sex, age, age of onset of AS, marital status, education level, occupation (full-time or part-time and whether AS affected work), presence of extra-articular manifestations, and history of falling within the past one year. Marital status was categorized as either married or other, which included single, divorced, separated, or cohabitation. Education level was categorized as either senior high school and below, determined by the 12-Year Basic Education policy of the Ministry of Education [33], or college and above.

#### Disease activity

The Bath Ankylosing Spondylitis Disease Activity Index (BASDAI), designed by Garrett (1994) [34], was used to measure the status of AS within the preceding week. The BASDAI is a 6-item questionnaire regarding fatigue, spinal pain, joint pain, enthesitis, duration of morning stiffness duration, and severity of morning stiffness. Questions scored on a visual analogue scale (VAS) of 0 (none) to 10 (very severe). The two scores for morning stiffness are averaged, added to the scores for questions the other four questions, and the sum is divided by five for the final score; a higher score indicates a higher level of disease activity. Scores of 0–2 indicate mild disease activity, 2.1–4.9 indicate moderate disease activity, and scores of 5 or higher indicate severe disease activity [9]. We used a Chinese version of BASDAI, developed and validated by Wei et al. (2007) for patients with AS in Taiwan. The Cronbach’s α was 0.87 [35]; in this study the Cronbach’s α was 0.93.

#### Daily functional activity

The Bath Ankylosing Spondylitis Functional Index (BASFI), developed by Calin et al. (1994) [36], was used to measure daily living function of patients with AS within the preceding week. The questionnaire consists of 10 questions concerning functional limitations of activities. The first 8 questions are related to anatomical limitations (such as bending, reaching); the last 2 questions involve the patients ability to cope with everyday life. Questions are scored on a VAS scale of 0 (easy) to 10 (impossible). The total BASFI score is the mean score of the 10 questions, which ranges from 0 to 10; a higher score indicates a higher level of functional impairment. A score of 0–2 indicates mild disability, 2.1–4.9 indicates moderate disability, and 5 and higher indicates severe disability. We used a Chinese version of the BASFI developed and validated by Wei et al. (2007) for use in patients with AS in Taiwan. The Cronbach’s α was 0.94 [35]; in the present study, the Cronbach’s α was also 0.94.

#### Pain

The numeric rating scale (NRS) was used to assess pain [37]. The NRS is a self-report scale which uses a VAS ranging from 0 (no pain) to 10 (worst pain ever); a higher number indicates more severe pain.

#### Body image

The body image scale (BIS) used in this study was developed by Huang (2013) for use in Taiwanese patients with rheumatoid arthritis; scores range from 0 to 100, with higher scores indicating a more positive body image. The scale has a Cronbach’s α of 0.91 [30]. In this study, the scale evaluated feelings, perceptions, and opinions of the patients with AS toward their bodies; the Cronbach’s α in the present study was 0.92.

#### Depressive symptoms

The 21-item Beck Depression Inventory-II (BDI-II) is a validated self-report questionnaire used to evaluate the of severity of depressive symptoms with questions related to emotions, perceptions and physical symptoms of depression [38]. A total score of 0–13 indicates no apparent symptoms of depression; 14–19 indicates mild depressive symptoms; 20–28 indicates moderate symptoms of depression; and 29–63 indicates severe symptoms of depression. The sensitivity and specificity of the scale are 81% and 92%, respectively. This study used the Chinese version of the scale (BDI-II-C) developed by Yeung et al. (2002) for Chinese Americans; the sensitivity and specificity of this scale are 79% and 91%, respectively [39].

### Statistical analyses

SPSS 22.0 for Windows was used to conduct statistical analysis. Descriptive analyses included frequency distribution, percentage, mean, and standard deviation (SD). A bivariate analysis was conducted using an independent sample *t* test and Pearson’s correlation. The significant predictive factors of depression were analyzed through multivariate linear regressions. The variables that exhibited a strong correlation with depression in the analysis results were identified as the predictive factors in the regression model according to the enter method; data independence, normal distribution, and multicollinearity were examined to confirm that these variables fulfilled the statistical hypotheses for a regression analysis. The level of significance was set at *p* < .05.

## Results

A total of 120 patients with AS met the inclusion criteria for participation in the study. The mean age of participants was 42.8 years (SD = 13.1); ages ranged from 20 to 74 years. Mean age at onset of AS was 29.1 years (SD = 11.2); mean duration of AS was 13.7 years (SD = 10.2). Most participants (68.3%, n = 82) were employed full-time; 57.5% (n = 69) reported their work had been affected by the disease. Extra-articular symptoms affected 54% of participants and 20 (30.9%) were diagnosed with ≥ 2 symptoms. A total of 14% of participants had fallen within the preceding year; of those, seven had fallen ≥ 2 times. Characteristics are shown in Table 1.

**Table 1:** Characteristics of the participants with AS (N = 120).

Characteristic	n	%	Mean	SD
Sex				
Female	29	24.2		
Male	91	75.8		
Age, years (Range: 20–74)			42.8	13.1
Duration of AS				
Age at onset, years (Range: 11–64)			29.1	11.2
Years since onset (Range: 1–50)			13.7	10.2
Marital Status				
Married	66	55.5		
Single or other	54	44.5		
Education				
High school or below	47	39.2		
College or above	73	60.8		
Employment status				
Unemployed or part time	38	31.7		
Full time	82	68.3		
Work affected by AS				
No	51	42.5		
Yes	69	57.5		
Extra-articular symptoms				
No	55	45.8		
Yes	65	54.2		
≥ 2 symptoms	20	30.9		
History of falls, within the past year				
No	103	85.9		
Yes	17	14.2		
≥ 2 times	7	41.0		

SD: standard deviation; AS: Ankylosing Spondylitis

Clinical variables for patho-physiological measures are shown in Table 2. Only a small number of participants exhibited abnormal CRP and ESR indices (n = 18 (15%) and n = 11 (9.2%), respectively). However, 64 participants (53.3%) exhibited measures of Cobb’s angle indicating moderate or severe kyphosis (mean = 23.10^o^; SD = 16.14). Slightly more than half of participants exhibited Level 3 or 4 dysfunction of the left or right sacroiliac joint (n = 65 (54%), n = 62 (52%), respectively).

**Table 2:** Mean scores for participants for clinical variables (N = 120).

	Scores for all participants				Participants with Abnormal/severe measures
Variable	Mean	SD	Median	Range	n (%), index
**Physio-pathological**					
CRP, mg/dl	0.62	1.23	0.23	0–8.19	18 (15%), ≥ 1.0 mg/dl
ESR, mm/hr	9.80	10.58	7.00	0–73.0	11 (9%), ≥ 20 mm/hr
Cobb's angle, degrees	23.10	16.14	23.00	0–70	64 (53.3%), moderate/severe
Sacroiliac joint severity					
Left	2.84	0.91	3.00	0–4	65 (54.2%), level 3 or 4
Right	2.81	0.91	3.00	0–4	62 (51.6%), level 3 or 4
**Self-report**					
BASDAI	2.90	2.01	2.45	0–8.4	43 (35.8%) - 23(19.2%), moderate—severe
BASFI	2.27	2.27	1.32	0–8.2	29 (24.2%) - 22(18.3%), moderate—severe
Depression (BDI-II)	9.50	8.30	8.00	0–42	30 (25.0%), mild—severe
Pain (NRS)	3.47	2.09	3.00	0–10	13 (11.7%), severe pain (7–10 points)
Body Image (BIS)	68.17	16.14	68.00	31–100	

SD: standard deviation; CRP:C-Reactive Protein; ESR: Erythrocyte sedimentation rate; BID-II-C, Chinese

version of the Beck Depression Instrument-II; ≥14 = mild to severe depression; BIS, Body Image Scale

BASDAI, Bath Ankylosing Spondylitis Disease Activity Index; BASFI: Bath Ankylosing Spondylitis Functional Index

NRS: Numeric Pain Rating Scale; BIS: Body Image Scale

Self-report measures of clinical variables (Table 2) showed 23 participants (19.2%) exhibited severe disease activity, as measured with the BASDAI. Scores on the BASFI indicated 22 participants (18.3%) exhibited severe functional disability. Scores on the BDI-II were ≥14 for 30 participants (25%), indicating mild to severe depressive symptoms; of these, 16 (13.3%), 9 (7.5%), and 5 (4.2%) had scores suggesting mild, moderate, and severe depressive symptoms, respectively. Although the mean level of pain was low (3.47), 13 participants (11.7%) experienced levels indicating severe pain (7–10 points).

### Depressive symptoms and characteristics of participants

To examine what characteristics of patients with AS might be associated with depressive symptoms, we first compared mean scores on the BDI-II with participants’ characteristics using independent samples t tests (Table 3). The mean score on the BDI-II was significantly higher for participants who were unemployed or employed part time (*p* < .001); those whose work had been affected by AS (*p* < 0.001); those with extra-articular symptoms (*p* = .002); and those who had fallen in the preceding year (*p* < .001).

**Table 3:** Mean scores for the Beck Depression Inventory-II and variables of participants (N = 120) with AS; comparison by independent samples t test.

Variable	Mean	SD	t	*p-*value
Sex				0.88
Male (n = 91)	9.44	8.22	-0.14	
Female (n = 29)	9.69	8.66		
Marital status				0.16
Married (n = 66)	8.55	7.97	-1.39	
Single or other (n = 54)	10.67	8.60		
Education				0.26
High school or below (n = 47)	10.55	8.19	8.35	
College or above (n = 73)	8.82	8.35		
Employment status				< .001
None or part time (n = 38)	13.61	8.75	3.90	
Full time (n = 82)	7.60	7.39		
Work affected by AS				< .001
No (n = 51)	5.69	5.12		
Yes (n = 69)	12.32	9.06	4.69	
Extra-articular manifestations				.002
No (n = 55)	7.11	5.35		
Yes (n = 65)	11.52	9.73	3.13	
History of falls				< .001
No (n = 107)	8.42	7.32		
Yes (n = 17)	16.06	10.79	3.70	

SD: standard deviation; AS: Ankylosing spondylitis

We examined mean scores for demographic and clinical characteristics associated with AS for participants and mean scores for depressive symptoms with Pearson’s correlation coefficient (Table 4). There was a significant positive correlation between depressive symptoms and severity of the sacroiliac joint (p < .01) and mean scores on the BASDAI and BASFI (p < .001) well as the NRS (p < .01). Mean scores for the BIS were negatively correlated with depressive symptoms (*p* < .001).

**Table 4:** Pearson’s correlations for demographic and clinical variables for participants with ankylosing spondylitis and mean scores for depressive symptoms (N = 120).

Variable	r	p-value
Age	0.05	0.536
Age at onset	-0.09	0.314
Years since onset	0.18	0.055
Right sacroiliac joint severity	0.26	0.004
Left sacroiliac joint severity	0.27	0.002
Cobb's angle	0.13	0.164
CRP	0.04	0.638
ESR	0.03	0.720
BASDAI	0.62	< .001
BASFI	0.62	< .001
NRS	0.24	.009
BIS	-0.68	< .001

CRP:C-Reactive Protein; ESR: Erythrocyte sedimentation rate; BASDAI: Bath Ankylosing Spondylitis Disease Activity Index; BASFI: Bath Ankylosing Spondylitis Functional Index; NRS: Numeric Pain Rating Scale; BIS: Body Image Scale

Multiple linear regression was used to determine what factors significantly correlated with depressive symptoms in patients with AS. Associated factors were identified using the enter method (Table 5). The mean score for BASDAI (*p* = .032), effect of AS on work (*p* = .049), occurrence of a fall within the preceding year (*p* = .032) and BIS (*p* < .001), were determined to be key associated factors for depressive symptoms. The regression model exhibited an explanatory power of 57% (adjusted R^2^ = .57).

**Table 5:** Predictors of depression: Multiple linear regression; Enter model (N = 120).

						95% CI for *B*	
Variable	*B*	*SE*	*β*	t	*p-value*	Lower bound	Upper bound
Occupational Status (1 = Full time)	-2.0	1. 21	-0.12	-1.69	0.093	-4.44	0.35
Work affected by AS (1 = Yes)	2.31	1.16	0.14	1.99	0.049	0.01	4.60
Extra-articular manifestations (1 = Yes)	0.87	1.08	0.05	.81	0.420	-1.26	3.00
History of falling (1 = Yes)	3.30	1.52	0.14	2.17	0.032	0.28	6.31
Right sacroiliac joint severity	-0.32	1.72	-0.02	-0.19	0.854	-3.73	3.09
Left sacroiliac joint severity	-0.23	1.66	-0.01	-0.14	0.891	-3.52	3.06
BASDAI	0.86	0.40	0.21	2.17	0.032	0.08	1.64
BASFI	0.54	0.37	0.15	1.49	0.140	-0.18	1.27
NRS	-0.21	0.34	-0.05	-0.63	0.528	-0.88	0.45
BIS	-0.20	0.04	-0.38	-4.44	< .001	-0.28	-0.11
*R*^*2*^ = 0.61							
*Adjusted R*^*2*^ = 0.569							

AS: Ankylosing Spondylitis; BASDAI: Bath Ankylosing Spondylitis Disease Activity Index; BASFI: Bath Ankylosing Spondylitis Functional Index; NRS: Numeric Pain Rating Scale; BIS: Body Image Scale

## Discussion

The findings from this study indicated that 25% of participants had scores on the BDI-II indicating mild or greater levels of depressive symptoms; this percentage is higher than that reported in an investigation of the global prevalence of depression conducted by the World Health Organization [40]. However, this percentage is similar to that reported in other studies on depression in patients with AS [14, 16].

A small percent of the participants with depressive symptoms had scores suggesting moderate to severe depressive symptoms (11.7%). Several possible bio-physio-psycho-social mechanisms might explain the increased risk of depressive symptoms in patients with AS, including the association of depression with a chronic, low-grade inflammatory response and activation of cell-mediated immunity [6, 41]. AS is a chronic inflammatory disease, and this multi-systemic inflammatory disorder stimulates proinflammatory cytokines, which may induce and increase depressive symptoms [6, 41, 42]. TNF-alpha inhibitors act as disease-modifying antirheumatic drugs (DMARDs), which have been shown to improve auto-immune and inflammatory symptoms as well as depressive symptoms in patients with AS [17, 42]. Genetics also may play a role in AS in terms of disease susceptibility, and the influence of HLA-B27, and inflammatory genes, such as IL23R, ERAP1, and interleukin-1α, and the presence or absence of depressive symptoms [6, 43]. Psycho-social stresses have also been associated with depressive symptoms in patients with AS, which include loss of work productivity and body image disturbance [7, 44]. These bio-physio-psycho-social mechanisms associated with depressive symptoms are complex. Therefore, comprehensive assessment related to depressive symptoms should evaluated during routine exams and long-term follow-ups. This could be achieved by incorporating a multidisciplinary professional team into the evaluation and intervention in order to simultaneously review the use of DMARDs, as well as physical, psychological, and social support for patients with AS [6, 42].

Body image was significantly associated with depressive symptoms for participants in this study. Body image involves the interaction of neurosensory systems, psychological perceptions, and social aspects [7, 9]. Although extra-articular symptoms, sacroiliac joint severity, and Cobb's angles (severity of kyphosis) were not significantly associated with depressive symptoms, a recent study found these variables to be negatively correlated with body image for patients with AS [45]. A disturbance in body image occurs when individuals are unable to accept changes in their bodies; this results in a lower sense of self-worth, which can lead to depression and anxiety [7, 46, 47]. Compared with healthy individuals, patients with AS are more concerned with potential body flaws, have a lower self-confidence in their attractiveness and general appearance, and higher levels of impaired interpersonal relationships [9, 48]. Patients with AS experience uncertainty regarding disability and prognosis; this uncertainty can cause anxiety, undermine patients’ perceptions of their bodies, and increase the risk of depression [8, 46, 48]. Clinical professionals should emphasize disease management, including physiotherapy, as a means of enabling patients with AS to manage the impact of this disease on their physical and psychological health, and reduce deformities of the spine. Moreover, patients must be provided with psychological support to enable them to accept the disease and develop strategies for adapting to it, thereby mitigating the effect of negative body image on their psychological health.

BASDAI score was determined to be a significant factor for depressive symptoms in patients with AS, which is consistent with the findings of relevant studies; patients with higher scores on disease activity exhibited higher scores for depression [7, 8, 49]. BASDAI and BASFI scores, which are used to measure the disease activity and physical impairment of patients with AS, have been identified as critical factors associated with depressive symptoms of patients with AS in most systematic studies [14, 16]. In the present study, however, although both BASDAI and BASFI scores were significantly correlated with depressive symptoms, only the BASDAI was significantly associated with depressive symptoms. One explanation may be the BASDAI includes patients subjective perceptions of morning stiffness, pain, and fatigue which may be factors that exacerbate their depressive symptoms [13, 14, 25]. Clinical workers should heed the effect of disease activity on depressive symptoms in these patients. Although no medical cure for AS currently exists, effective disease self-management [50], exercise, and rehabilitation intervention programs have been proven to mitigate the disease activity of patients with AS and maintain or improve their spinal function and capacity for physical activity [51–53]. These programs should be implemented in clinics to prevent exacerbation of the disease and ameliorate patients’ depressive symptoms.

A history of falling was also a significantly associated with depressive symptoms. Of the participants in the present study, 14% had fallen within the preceding year, which is similar to findings of another study reporting 13% of patients with AS had fallen [53]. Patients with AS are at higher risk of falling than healthy people because of their limited spinal mobility, adhesive tendons, ligaments, and joints, diminished lower limb stability and poor posture [25, 53]. Indeed, a small fraction of participants had fallen ≥ 2 times in the preceding year. Falling can exacerbate disabilities, and falling repeatedly can also damage a patient’s self-confidence and increase the development of depressive symptoms [25]. Although we did not collect data regarding the presence of osteoporosis in our participants, it is a common comorbidity in patients with AS and can lead to bone fractures, which can increase fracture severity when patients fall [54, 55]. Therefore, balance and gait training, fall prevention, and safety protection must be implemented in AS care programs.

Participants who were unemployed and whose work had been affected by AS scored significantly higher for depressive symptoms than those who were employed and whose work capacity had not been affected, which is consistent with findings reported in other studies [23, 56, 57]. Work disability and unemployment are closely associated with depressive symptoms. Patients with AS, particularly those whose employment involves physical labor, are more prone to work disability than healthy individuals [23]. Nearly 60% of the participants reported that their work had been affected by AS and most participants in the present study were of working age. Therefore unemployment and limitations to career development would affect not only their personal economic status, but also that of their family, which impacts psychological health and social relationships [22, 56]. Work-related problems should be routinely discussed with patients with AS during clinical assessments, which should include effects of disease activity and fatigue [21]. Occupational redesign and the promotion of friendly work environments should be reinforced to improve the ability of these patients to adapt to the workplace. Flexible work schedules, exercise, and rehabilitation must be implemented to prevent these patients from premature unemployment and thereby reduce their risk of depressive symptoms.

Female patients with AS are at higher risk of depressive symptoms than males [11, 14, 40]. However, sex was not an associated factor in this study. One possible explanation is females represented only 26% of the participants in our study. However, a meta-analysis by Zhao et al. [14], suggested levels of depression may be overestimated, since depression in the general population is higher for females than males. Our findings also differed other studies that have shown correlations between educational level and depressive symptoms [10, 19]. This finding might be explained by the educational level of participants in this study; 61% had at least a college education.

### Research limitations

Our findings might be limited by the small sample size of participants (N = 120) from one medical center in Taiwan, and the exclusion of patients with additional disease complications. Therefore, our results might not be representative of the entire population of individuals with AS, which might limit generalization of our findings. In addition, we did not collect information about the participants’ previous clinical diagnoses of depression, other depression-related psychiatric disorders, or use of antidepressants and DMARDs; we also did not obtain information about comorbid auto-immune diseases. As these variables have been shown to influence outcomes [6, 42], future studies should be conducted that control for these factors. Although this study investigated whether AS affected participant employment, it did not examine the specific employment aspects that AS affected; therefore, the obtained results should be interpreted with caution. The temporal nature of this cross-sectional correlational study provided only data regarding depressive symptoms in patients with AS at a single point in time. A patient’s psychological status varies with changes in physical and mental health, as well social relationships. Therefore, in order confirm the effect of disease conditions on depressive symptoms in patients with AS, more rigorous studies should be conducted. Long-term studies with control populations that include assessments for physical, mental, and social variables could strengthen our findings.

## Conclusions

Factors that might contribute to depressive symptoms in patients with AS should be explored during clinical assessments. The variables identified as being significantly associated with depressive symptoms for participants in this study included body image, disease activity, occurrence of falling within the preceding year, and effects of AS on work. A multidisciplinary cooperative approach to patient care with regular assessments of these factors associated with depressive symptoms should be incorporated into routine care programs to enhance self-care capabilities, and prevent or mitigate depressive symptoms for patients with AS. Long-term follow-ups which include physical, mental, and social aspects associated with depressive symptoms for patients with AS should be considered in clinical practice and in future studies.

## Supporting information

S1 File Dataset. (XLSX)
